# Supplementary material for: Impact of habitat loss on the diversity and structure of ecological networks between oxyurid nematodes and spur-thighed tortoises (Testudo graeca L.)
Source: PeerJ. 2019 Dec 2;7:e8076. doi: 10.7717/peerj.8076 (PMC6894431; doi:10.7717/peerj.8076)
Supplement: Supplemental Information 1 — The calculation of individual tortoise (Testudo graeca) growth rate (mm year−1, k) using the von Bertalanffy model. [file peerj-07-8076-s001.docx]

***Appendix 1. Growth rates***

Individual growth can be assessed by the development of growth models. This method is particularly suitable for indeterminate growth, which is the case for tortoises because they grow throughout their lifespan. Growth in adults, however, is asymptotical towards maximum size. The von Bertalanffy model is the most commonly used growth model for explaining the individual growth of tortoises (Equation 1; Zivkov et al. 2007; Macale et al. 2009, Rodriguez-Caro et al., 2013).

Equation 1

| $Y= S_{\infty}$(1 - b$e^{-kt}$) |  |
| --- | --- |

where the dependent variable *Y* is carapace length at age *t*, $S_{\infty}$ is asymptotic size, *b* is a parameter associated with the amount of growth remaining, and *k* is the growth rate (Fabens 1965). In Rodríguez-Caro et al. (2013), the parameters $S_{\infty}$, *b* and *k* were estimated using nonlinear regressions between captures and recaptures.

The growth rate *k* is a parameter that describes the process of growth from the birth of the individual to the present. To know the variability in *k*, we used the size of each individual *Y* and the estimated age by growth ring counting *t* (Rodríguez-Caro et al., 2015). We estimated *k* for each individual using Equation 2 and the previously estimated values of $S_{\infty}$ (172 mm and 131 mm for females and males, respectively) and *b* (0.7843 and 0.7112 for females and males, respectively).

Equation 2

| $k= \frac{\ln\left( -\frac{\frac{Y}{S_{\infty}}-1}{b} \right)}{-t}$ |  |
| --- | --- |

The distribution of growth rates ranged from 0.0019 to 0.4559, with a mean of 0.1095 (Fig S1.1). There were no differences in growth rates between landscapes with different habitat loss categories (Fig. S1.2.).


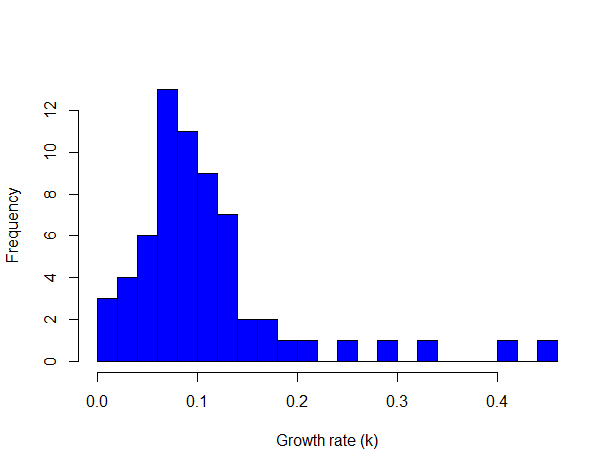


**Fig S1.1. Distribution of individual growth rates.** Histogram with the values of *k*.


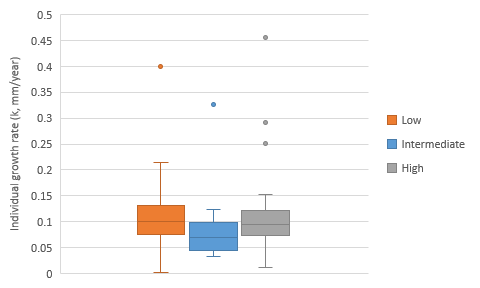


**Fig S1.2. Individual growth rates in landscapes.** Box plot with the values of *k* according to the level of habitat loss.

**References**

Fabens AJ. 1965. Properties and fitting of the von Bertalanffy growth curve. Growth 29:265-289 https://www.cabdirect.org/cabdirect/abstract/19661402866.

Macale D, Scalici M, Venchi A. 2009. Growth, mortality, and longevity of the Egyptian tortoise Testudo kleinmanni Lortet, 1883. Israeli Journal of Ecology and Evolution 55: 133-147 doi.org/10.1560/IJEE.55.2.133

Rodríguez-Caro RC, Gracía E, Anadón JD, Giménez A. 2013. Maintained effects of fire on individual growth and survival rates in a spur-thighed tortoise population. European Journal of Wildlife Research 59:911-913 doi.org/10.1007/s10344-013-0764-1.

Zivkov M, Ivanchev I, Raikova-Petrova G, Trichkova T. 2007. First data on the population structure, growth rate and ontogenetic allometry of the tortoise *Testudo hermanni* in eastern Stara Planina (Bulgaria) *- Comptes Rendus de L’Academie Bulgare des Sciences* **60:**1015-1022
